# A Novel Biopsy Capsule Robot Based on High-Speed Cutting Tissue

**DOI:** 10.34133/2022/9783517

**Published:** 2022-08-05

**Authors:** Zhibin Song, Wenjie Zhang, Wenhui Zhang, Dario Paolo

**Affiliations:** ^1^ Key Laboratory of Mechanism Theory and Equipment Design of the Ministry of Education, Tianjin University, Tianjin 300072, China; ^2^ Beijing Shijitan Hospital, Capital Medical University, Beijing 100084, China; ^3^ The BioRobotics Institute, Scuola Superiore Sant’Anna, 56100 Pisa, Italy

## Abstract

The capsule robot (CR) is a promising endoscopic method in gastrointestinal diagnosis because of its low discomfort to users. Most CRs are used to acquire image information only and lack the ability to collect samples. Although some biopsy capsule robots (BCRs) have been developed, it remains challenging to acquire the intestinal tissue while avoiding tearing and adhesion due to the flexibility of colonic tissue. In this study, we develop a BCR with a novel sampling strategy in which soft tissue is scratched with sharp blades rotating at high speed to avoid tissue tearing. In the BCR design, a spiral spring with prestored energy is used to release high energy within a short period of time, which is difficult for a motor or magnet to perform within a small capacity installation space. The energy of the tightened spiral spring is transmitted to drive sharp blades to rotate quickly via a designed gear mechanism. To guarantee reliable sampling, a Bowden cable is used to transmit the user’s manipulation to trigger the rotation of the blades, and the triggering force transmitted by the cable can be monitored in real time by a force sensor installed at the manipulating end. A prototype of the proposed BCR is designed and fabricated, and its performance is tested through in vitro experiments. The results show that the proposed BCR is effective and the size of its acquired samples satisfies clinical requirements.

## 1. Introduction

Colorectal cancer has become the third most common malignant tumor and the second most deadly cancer in the world. In 2020, it had an incidence of approximately 1.9 million cases and caused 0.9 million deaths [1]. The survival rate of stage I colorectal cancer can reach 90%, but the survival rate of stage V colon cancer is only 10% [2]. Therefore, early diagnosis of colon cancer can effectively reduce mortality.

Diagnosis of colorectal cancer mainly depends on endoscopy of the colon with a flexible and slender endoscope equipped with an image acquisition system, which unfortunately brings discomfort and risk to patients. In recent years, the capsule robot (CR) has emerged as an alternative to the traditional endoscope, as its small size can greatly decrease pain and mental stress experienced by patients [3, 4]. The movement of a typical CR is passive, meaning it depends solely on the peristalsis of the colon. As a result, CRs can sometimes miss lesions. Therefore, actively locomotive CRs have been developed. The trajectory of an actively locomotive CR can be modulated by an external magnetic field or internal actuators to achieve more thorough exploration of the colon. Both the position and posture of actively locomotive CRs could be controlled well via an external magnetic field [5–7], and images can be transmitted from CRs to an external receiver through a radio frequency (RF) transmitting circuit for real-time monitoring [8–10]. However, imaging is not sufficient for diagnosis, because suspicious lesions must be biopsied. Biopsies can be taken by a traditional endoscope but not a CR. This is where the biopsy capsule robot (BCR) comes in.

Already, scientists and engineers have developed many kinds of BCRs. Park et al. designed a microactuator integrated into a BCR, which consists of a spiral spring, a microspike, and a shape memory alloy (SMA). The microactuator can tear off and retain samples, while the microspike moves the BCR to and away from target tissues [11]. Chen et al. designed a micro biopsy device including a micro biopsy jaw and a micro-motor. The micro biopsy jaw can derive sufficient force from the micro-motor to implement biopsy, but the allocation of space for the motor is undesirable [12]. Le et al. designed a miniaturized biopsy module using a gripper driven by an external magnetic field. Although the biopsy module can precisely reach target lesions, it may tear the tissue during sampling because stable force can hardly be obtained by the external magnetic field [13].

Instead of jaws or grippers, Kong et al. proposed a micro BCR that uses a rotational tissue cutting razor for biopsy. The BCR also includes a spiral spring and a trigger with a paraffin block. When the paraffin block melts by heating, the razor is released to rotate and cut tissue. The problem is that the timing of the razor release cannot be controlled accurately via melting of the paraffin block [14, 15]. Kong et al. also introduced a BCR including a tissue monitoring module to monitor the desired zone for biopsy, an anchor module, and a biopsy module. The biopsy module includes two cylindrical razors triggered by melting of a chip resistor with electric power [15].

A magnetic field was used as the energy source to control the movement of an actively locomotive BCR and power the biopsy [16, 17]. Both studies reported that it was challenging to reliably and simultaneously achieve both locomotion and biopsy control for the BCR due to mutual disturbance during the magnetic field. Simi et al. introduced a BCR that includes a magnetic spiral spring mechanism composed of two coaxial cylindrical diametrically magnetized permanent magnets. Biopsy is triggered by an external permanent magnet combined with a couple of fixed and freely rotated cylindrical permanent magnets. This solution allows for miniaturization since no batteries and no control electronics are required on board, but the remote detection of the open and closed state of the mechanism lacks a sensing system [18].

In addition, some novel sampling methods have been developed for BCRs. Yim et al. proposed a magnetic BCR that can release micro-grippers (u-grippers) and retrieve them after they have self-folded to grab tissue samples [19]. Son et al. proposed a magnetically actuated soft BCR that takes biopsy samples using a fine-needle biopsy technique [20]. The above methods can collect sampling tissue. However, the micro-grippers are limited by their low rate of retrieval, and the soft robotic capsule is limited by its mode of locomotion (i.e., rolling only) [17].

In fact, in practical clinic application, a scissor mechanism that can hold and cut tissue with closing jaws is widely used in biopsy forceps [21]. However, tissue tearing sometimes happens when jaws are not sharp enough or are not closed completely. The scissor mechanism is not suitable to be used by BCRs. Instead, sharp, rotating blades are often used. Previous studies have reported many solutions for taking biopsies in ideal conditions. Practically, though, the interaction between BCR and colon is complex due to the flexibility of the colon, which may increase the difficulty in biopsy.

In this study, we develop a novel BCR base on the high-speed cutting tissue in which a cutter composed of sharp blades is rotated at high speed to cutting the tissue of the colon. This BCR can achieve effective and reliable sampling because the cutting force that the force required by the cutter to cut the soft tissue is low and the tissue deformation is small. A small permanent magnet is installed in the BCR, and the locomotion of the BCR can be controlled by the external magnetic field. A high-speed cutter in the BCR is activated by a tightened spiral spring because such springs can provide greater power than motors or magnets with the same mass, which can effectively avoid mutual interference between the locomotion and biopsy control. A trigger mechanism was designed to guarantee reliable sampling, a Bowden cable is used to transfer the trigger force that the force of Bowden cable to trigger biopsy. To avoid spurious triggering, the trigger force is kept larger than a specified threshold, and it can be monitored in real time by a force sensor installed at the manipulating end. A prototype of the proposed BCR with this novel sampling strategy is fabricated, and its biopsy performance is proven through in vivo experiments.

This paper is organized as follows. The working principle and the structural design of the BCR and the trigger force model are introduced in Section 2. Fabrication and assembly of the prototype are detailed in Section 3, and experimental assessment is reported in Section 4. A discussion of our work and our conclusions are presented in Section 5.

## 2. Materials and Methods

### 2.1. Structural Design and Working Principle of the Proposed BCR

As is well known from experience with cooking, it is easy to cut meat with a sharp knife when the meat is on a hard chopping board; however, it would be much more difficult to cut the meat if the meat was hanging. Similarly, cutting intestinal tissue with a blade held by a BCR is difficult because the tissue is very flexible and the BCR cannot be fixed stably. Based on the idea above, we proposed a new sampling strategy in which sharp blades are rotated at high speed to cutting suspicious areas of the colon. During this process, the cutting force decreases, while the rotational speed of the blade is very high, and the edge of the blade is very sharp so that the position of the BCR relative to the colonic tissue can merely be influenced. Furthermore, to avoid disturbance from the external magnetic field used to drive the locomotion of the BCR and also to keep the BCR compact, a compressed spiral spring is used to power the blades. Finally, to implement reliable triggering of sampling, a Bowden cable mechanism is used, and the tension in the cable can be monitored in real time.

**Figure 1:**
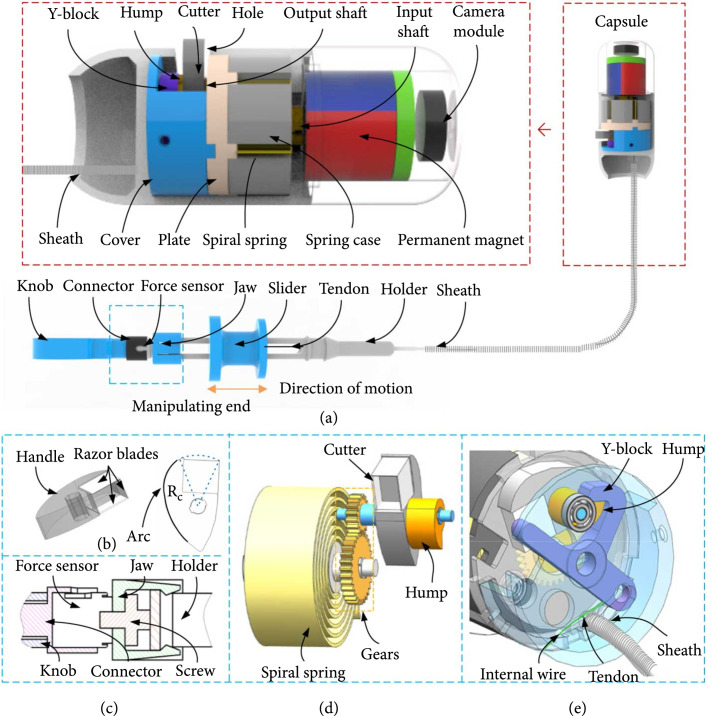
(a) Structure of biopsy capsule robot. (b) Structure of cutter. (c) Structure of buckle. (d) Diagram of transmission. (e) Diagram of trigger device.

According to the proposed method, a BCR system is designed to realize the abovementioned functions. The structural design is shown in Figure 1(a). The system is composed of a capsule and a manipulator, which are connected to each other by a Bowden cable (sheath and tendon). The capsule consists of a biopsy part, a permanent magnet and the camera module. In the biopsy part, a tightened spiral spring is installed in the spring case. The spring’s stored elastic energy is transmitted via a gear mechanism with a designated transmission ratio to power a rotational cutter composed of razor blades to perform biopsy, as shown in Figure 1(d). The hump in the output shaft and the Y-block constitute a switch to control the release of the spiral spring. The biopsy is performed outside of the capsule, as the cutter travels out of the capsule through a long hole on the shell of the capsule. To achieve reliable sampling, three sharp blades are fixed on a handle to form a rectangle cutter (Figure 1(b)), which can completely cut and store tissue. On the back of the handle, a circular arc structure is designed to seal the hole of the capsule shell to prevent fluid from entering when sampling is not being performed. A radial magnetized ring permanent magnet is fixed at the front of the capsule, and its N pole is on the same side as the hole. In addition, a camera module is installed on the front of the capsule.

**Figure 2:**
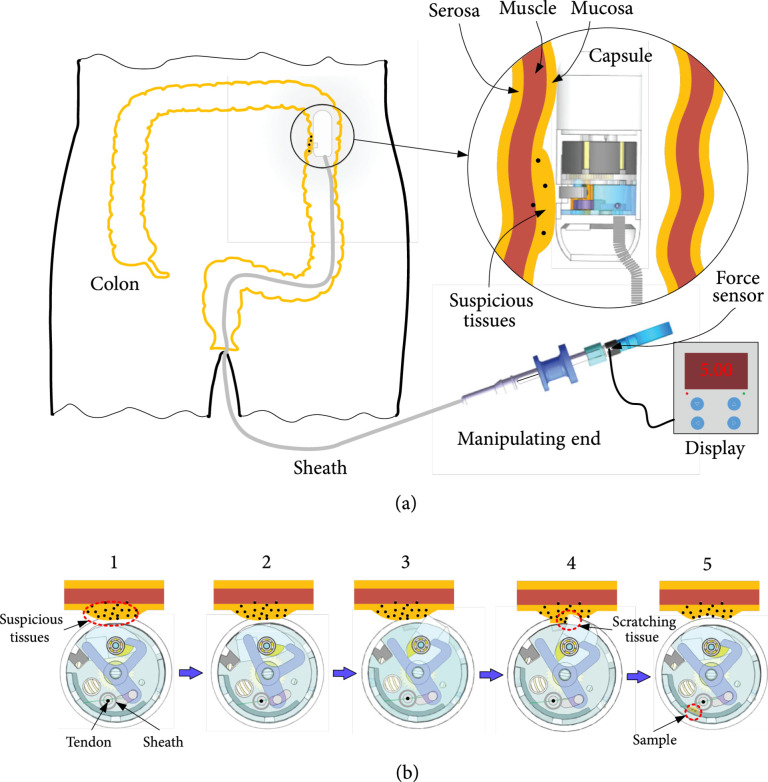
(a) Working scenario of BCR. (b) Working principle diagram of the biopsy process.

To realize reliable triggering, a self-resetting trigger is designed as shown in Figure 1(e). The trigger is composed of a Y-block, a hump block, a Bowden cable, and an internal wire. The handle of the cutter is fixed on the hump block through a shared shaft, which is the output of the gear mechanism. The hump block cannot rotate even if torque is applied to it via the gear mechanism, while it is blocked by the Y-block claw, as shown in Figure 1(e). The internal wire connects one end of the Y-block and the cover. The wire can be pulled by the Bowden cable, which makes the Y-block rotate around its shaft, and the cutter along with the hump block can be released and can rotate quickly (Figure 2(b)). The rotating block hits the opposite edge of the Y-block claw to reset the Y-block (Figure 2(b)3) and then is stuck by the claw of the Y-block again (Figure 2(d)). This trigger mechanism design can realize the effective position limit of rotational cutter under one trigger.

To make it easy for the operator to trigger biopsy, the manipulator connected to the capsule through the Bowden cable is utilized. In the manipulator, the slider connected to the tendon in the Bowden cable can slide along the holder to pull the internal wire. The manipulator has an embedded force sensor, which can record the pressure between the slider and the holder, when the slider slides along the holder. Considering the convenient connection, an easy plug-in design is adopted in the manipulator, as shown in Figure 1(c). The holder is inserted into the jaw connected to the force sensor, and the claws on the edge of the jaw can easily clamp the holder to realize the connection. According to this design, the triggering force transmitted to the manipulator via the Bowden cable can be measured by the force sensor.

The working scenario of the BCR is shown in Figure 2(a). The BCR enters the colon from the anus, and once the BCR approaches the suspicious tissue, the tendon of the Bowden cable can be pulled by the slider in the manipulator to trigger sampling. The trigger force can be monitored via a display panel and compared to a specified threshold to ensure reliable trigger. To enable active locomotion, the BCR contains a small permanent magnet, which can be controlled by external magnetic field. This paper focus on the biopsy of BCR; the magnetic driving principles are beyond the scope of this article. More detailed external magnetic driving principles are implemented according to those studies [7–10]. The specific working process of biopsy, shown in Figure 2(b), is as follows:

(1) The posture of the capsule is adjusted—through control via an external magnetic field—so that its hole is close to the inner wall of the suspicious area of the colon. At this time, the tightened spiral spring in the capsule retains its stored energy and the output shaft of the cutter and the gear mechanism remain in equilibrium status since the output shaft is constrained by the Y-block
(2) A user manipulates the slider to pull the tendon of the Bowden cable to drag the internal wire, which drives the Y-block to rotate clockwise (Figure 2(b)). Due to the rotation of the Y-block, the claw of the Y-block releases the hump; so, the hump and the cutter begin to rotate. During this process, the trigger force is monitored and shown on a digital panel
(3) The cutter fixed on the output shaft exits the shell hole and rotates rapidly to cutting the suspicious soft tissue (Figure 2(b)4). The scratched-off tissue is flung inside the cutter for storage. Then, the rotating block hits the opposite edge of the Y-block claw to reset the Y-block
(4) Finally, the rotating hump block is stuck by the claw of the Y-block, and the soft tissue in the cutter is thrown onto the bottom of the cover area due to its inertia
(5) After a biopsy is completed, a user can pull the tendon of the Bowden cable again to collect samples from the same part. The different targets can also be sampled under the control of external magnetic field

### 2.2. Analysis of Cutting Force

In order to cut flexible tissue effectively, the cutter needs to have sharp blades and provide sufficient cutting force. Razor blades are of absolute sharpness so that they can be used to cut tissue well. From the perspective of mechanics, the shear stress of the blade of the cutter should be greater than the destruction stress of the tissue. According to the research of Kong et al. [15] and Simi et al. [22], the destruction stress of the colon tissue is about 20 Mpa; so, the shear stress at the blade edge of the cutter should be greater than 20 Mpa when cutting the tissue. Shear stress can be calculated using the following formulas:

(1)τd=Ftw,(2)Mo=FRc,(3)Mi=Mo/iη,

where $\tau_{d}$ is the shear stress, F is the cutting force actuated by the cutting blade, which is related to the stiffness of the soft tissue and the speed of the cutter, t is the thickness of the cutting blade, w is the width of the cutting blade, $M_{o}$ is the cutting torque of the tissue, $R_{c}$ is the maximum radius of rotation of the cutter, $M_{i}$ is the input torque of the spiral spring, i is the transmission ratio of the gear transmission system, and η=0.9 is the gear transmission efficiency. If the thickness of the blade is very large, the cutting force will be very large, resulting in excessive energy consumption. Therefore, the thickness should be as small as possible. The width of the blade should also be selected appropriately, because a large width will increase its size, and a small width will lead to small tissue samples unsuitable for diagnostic tests.

Although the preceding method can determine the static cutting force, it does not account for the effect of speed on the cutting force. When a high-speed rotating tool cuts soft tissue, the soft tissue deformation is small, the tool’s effort is minimal, and the cutting force required for soft tissue cutting is minimal. Equation (4), which expresses the relationship between cutting force and cutting speed, is obtained by evaluating the cutting process in order to characterize the impact of tool rotation speed on cutting force. It can be found that the cutting force is related to the speed and rotational inertia of cutter. The process of cutting soft tissue with a cutter is difficult to observe; so, the specific duration of cutting soft tissue cannot be recorded accurately. We calculate the duration of soft tissue cutting by calculating the average cutter speed and cutter rotation angle as shown in Equation (5):

(4)FsRcT=Jω2−Jω1,(5)FsRcφωc=Jω2−Jω1,

where $F_{s}$ is the cutting force considering the speed, $R_{c}$ is the maximum radius of rotation of the cutter, T is the time for the cutter to cut the soft tissue, J is the moment of inertia of the cutter, $\omega_{1}$ and $\omega_{2}$ are the angular velocities before and after the soft tissue is scratched, respectively, $\omega^{c}$ is the average angular velocity while cutting the soft tissue, which can be expressed as $\left(\omega_{1}+\omega_{2}\right)/2$, and φ is the rotation angle of the cutter while cutting soft tissue.

### 2.3. Analysis of Trigger Module

**Figure 3:**
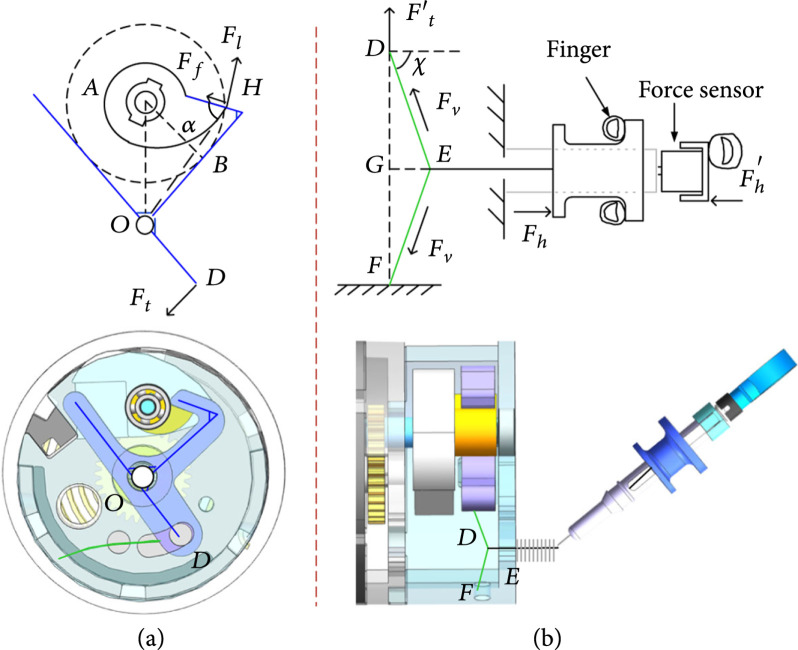
(a) Principle diagram of trigger forces. (b) Schematic diagram of operating handle trigger.

A reliable trigger module is very important for a BCR. For the proposed BCR, the core component of the trigger module is spring energy storage and release conversion. The trigger module can endure the initial tension by the loaded spring and can release quickly when a user pulls the tendon of the Bowden cable. Pulling the internal wire releases the trigger, which can translate cable strain into Y-block rotation, as illustrated in Figure 1(e). The trigger will not be triggered if the trigger force supplied by the Bowden cable is insufficient. To ensure a dependable trigger, we study the trigger process and build a statics model. The arc trajectory of the rotating end of the hump block illustrated in the orange dotted circle is tangent to two edges of the Y-block in Figure 3(a). The Y-block clamps the hump block in its original position. In Figure 3(b), DF refers to the internal wire that connects one end to the Y-block and the other end to the cover. The internal wire on midpoint E of the DF is linked by the Bowden cable. The tendon fixed on the slider pulls on the internal wire, causing the Y-block to spin and release the cutter. The tension in the cable can be monitored by the sensor in the manipulator. To complete the trigger, the tension must overcome the hump-generated frictional resistance of the Y-block, and the frictional resistance is related to the output torque of the spiral spring. Therefore, the cable must provide sufficient force, and the minimum tension needs to satisfy Equation (7). By analyzing the mechanics shown in Figure 3, the following formulas are obtained:

(6)Ff=μFlFflOHsinα=FtlODcos2χ=lED2+lEF2−lFD22lEDlEF,(7)Ft′<Fh2cosχsinχ,(8)Mo=Fl′lAH=kθ2i2,

where μ is the friction coefficient; α is the angle between the line OH and the hook of Y-block; χ is the angle between the cylindrical axis and the line ED; $l_{OH}$, $l_{OD}$, $l_{ED}$, $l_{EF}$, $l_{FD}$, and $l_{AH}$ are the lengths of relative segments; $F_{l}$, $F_{f}$, $F_{t}$, and $F_{h}$ are, respectively, the force exerted by the hump block on the Y-block, the frictional force between the hump block and the hook of Y-block, the force of the flexible internal wire on the Y-block, and the tension supplied by the cable; $M_{o}$ is the cutting torque of the tissue; k is the stiffness of the spring; i is the gear transmission ratio; and $\theta_{2}$ is the rotational angle of the output shaft. According to the above formulas, the reaction force exerted by the block on the Y-block as well as the minimum cable tension to launch the trigger can be calculated.

## 3. Prototype and Its Fabrication

**Figure 4:**
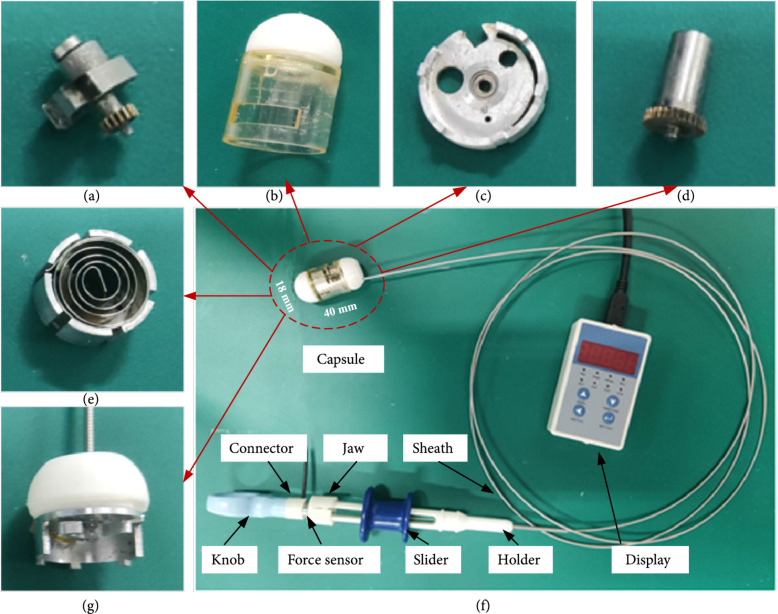
(a) Cutter and output shaft. (b) Shell. (c) Plate. (d) Input shaft. (e) Spiral spring installed in the housing. (f) Overview of BCR system. (g) Y-block and its cover.

In order to verify the feasibility of this scheme, we design and fabricate a prototype as shown in Figure 4. The spiral spring is made of a 304 stainless steel belt with 0.2-mm thickness. In order to ensure that the tissue can be scratched without being torn by the high-speed rotating cutter, the thickness of each blade (*t*) is 0.1 mm, and the width of the cutter (*w*) is 2 mm. So, the required cutting force is 4 N according to Equation (1). The razors are welded to the handle to make the cutter, and the maximum radius of rotation of the cutter ($R_{c}$) is 5 mm. The shell of the capsule is made by 3D printing technology. The two gears are made of brass by specific machining, and the numbers of teeth of the two gears are 30 and 16. The other parts are mainly made from aluminum alloy material using a CNC machining center. Finally, the designed capsule has a diameter of 18 mm and a length of 40 mm. The tendon-sheath mechanism is fabricated based on biopsy forceps (Yangzhou Fuda Medical Devices Co., Ltd). We adopted the tension transmission structure of commercial biopsy forceps as the tendon-sheath mechanism of our device via removing its part of jaw. The tendon was connected to the midpoint of the internal wire in capsule, and the sheath was fixed on the capsule end. When the tendon pulls the internal wire, the sheath prevents the BCR from sliding relatively. The knots were set on both sides of the midpoint of the internal wire to avoid relative sliding of the tendon. Furthermore, a force sensor (SBT-50 N) is installed on the manipulator via a 3D printing connector. A flexible jaw is designed to perform quick and easy connect between the force sensor and the holder so that the force sensor can be reused.

## 4. Results and Discussion

### 4.1. Experiments of Cutting Torque and Cutting Speed

**Figure 5:**
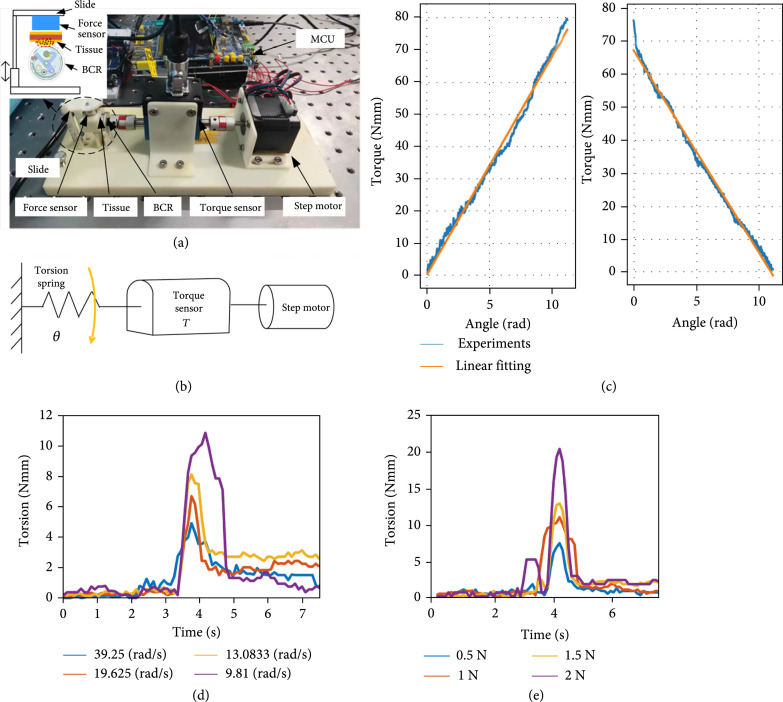
(a) Experimental setup for measuring the cutting speed and cutting torque. (b) Scheme of the experimental setup for measuring the spring torsion. (c) Experimental results for spring stiffness. The left graph shows the torque angle curve during the loading process of the spring, and the right graph shows the torque angle curve during the releasing process. (d) Cutting torque at different rotational speeds. (e) Cutting torque under different contact forces between the soft tissue and the capsule.

Equation (1) only provides the theoretical description of static cutting mechanics. The influence of the velocity of the cutter on the cutting torque that the torque required to cut the soft tissue on the axis of the cutter cannot be ignored, but it is difficult to describe the dynamic cutting mechanics owing to the complex mechanic characteristics of soft tissues. Therefore, we conduct experiments in order to obtain the relationship between the cutting torque and the speed of the cutter. As shown in Figure 5(a), a torque sensor (DYN-205) with 0.005 N·m resolution is adopted to detect the torque provided by the stepper motor to the input shaft of the capsule. Additionally, the contact force between the soft tissue and the capsule can also influence the cutting performance. In the experiments, this contact force is detected by using a force sensor (SBT-20 N). One side of the force sensor is mounted on a slide that can slide up and down, and the other side is in contact with the soft tissue. The force sensor’s position is adjusted to simulate the contact force between the capsule and the intestinal wall (Figure 5(a)). In the first experiment, the contact force is set as 0.1 N. Four different motor rotation speeds are set to drive the cutter to scratch the soft tissue, and the results are shown in Figure 5(d). When the motor speed is 9.81 rad/s, the maximum cutting torque measured by the torque sensor is 10.98 N·mm, which is less than the cutting torque calculated according to Equation (2). Moreover, we can also find that the faster the motor rotates, the smaller the cutting torque becomes. Therefore, the high-speed cutting method adopted in this paper can effectively reduce cutting torque. In addition, we also conduct experiments in which the motor speed is fixed at 9.81 rad/s, and the contact force between the capsule and soft tissue is adjusted. As shown in Figure 5(e), the greater the force between the capsule and the soft tissue, the greater the required cutting torque. The maximum cutting torque of 20.45 N·mm corresponds to the highest tested contact force because more tissues are squeezed into the cutting zone. This also means that larger tissue samples are acquired. In conclusion, the influence of velocity on cutting torque is determined through these experiments, and the results indicate that high-speed cutting can effectively perform biopsy with low cutting torque.

### 4.2. Measurement of Spiral Spring Stiffness

In the process of cutting soft tissue, a compressed spiral spring is adopted as the power source for the cutter. In order to ensure reliable cutting of soft tissues, the output torque of the spring should be higher than the damage stress of soft tissues. The mechanical properties of the spring will change after heat treatment, and the traditional calculation formula may not be applicable. In order to obtain the actual torque characteristics of the spring, the torque is tested in an experiment. The experimental platform is shown in Figure 5(b). The spiral spring is activated by the step motor, and the torque is measured by the torque sensor. Two experiments of spring loading driven by step motor and reversely releasing are carried out to measure the practical stiffness of the spiral spring. Figure 5(c) shows the experimental results of the output torque of the spring. We find that the torque-angle profile of the designed spiral spring approximately satisfies the linear relationship. From the results, the spring stiffness coefficient k=6.78 N·mm/rad is obtained. The maximum output torque of the spring can reach 70 N·mm/rad, which is greater than the 41.7 N·mm/rad calculated by Equation (3). The spiral spring can provide sufficient torque to complete biopsy. From the results of the releasing experiment, we find that there is an obvious decline in the beginning release process of the spiral spring, which is caused by a small gap between the fixed end of the spiral spring and the spring case.

### 4.3. Experiments of High-Speed Cutting Performance

**Figure 6:**
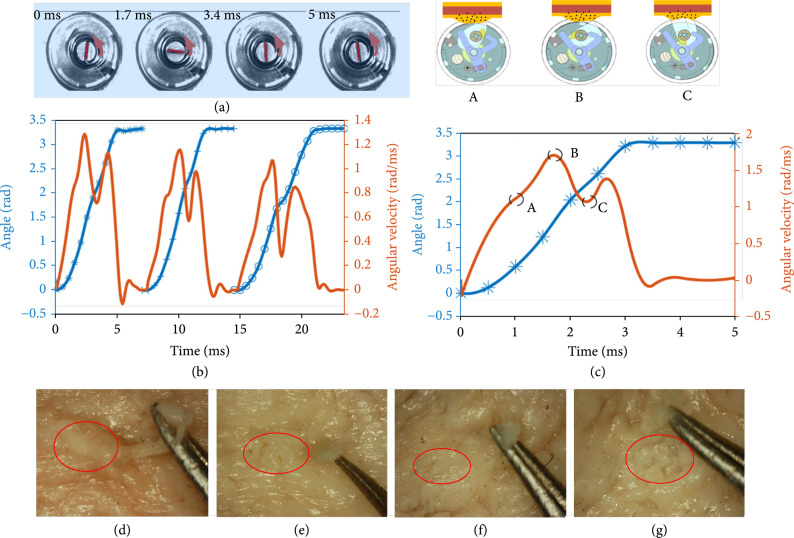
(a) Rotation of the spring during cutting of tissue in four recorded frames. (b) Rotating speed of the spring and the rotating angle of the cutter during the multiple biopsies process. (c) Rotating speed of the spring and the rotating angle of the cutter during the biopsy process. Point A indicates that the cutter slows down due to contact with soft tissue. Point B shows the critical point where the cutter begins to cut the tissue. Point C shows the critical point after the cutting is completed. (d)-(g) The result of sampling at cutting speed 0.5 rad/ms, 0.62 rad/ms, 1.05 rad/ms, and 1.5 rad/ms.

In order to analyze the cutting speed of the cutter driven by the spring in the cutting process, we also record the speed of the spiral spring during high-speed cutting of tissues through a high-speed camera (OSG030-815), which can be used to obtain the speed of the cutter by considering the gear mechanism. Through analyzing each frame, the rotational speed of the spiral spring can be calculated. Some typical frames are shown in Figure 6(a). The rotational angles and speed of the cutter are shown in Figure 6(c), from which we can see that the maximum speed during the cutting process reaches 1.71 rad/ms. From this figure, there are two important points before the cutter scratches the soft tissue. At point A, the end of the cutter extends out of the capsule and touches the tissue, which hinders the angular velocity of the cutter. At point B, the speed of the spring reaches the maximum value before the cutter begins to scratch the tissue, and then, the speed decreases during cutting. Although the end structure of the cutter will affect its rotation speed, the biopsy capsule can still cut the tissue at a high cutting speed without losing much speed. Therefore, this design can efficiently use high-speed cutting to achieve tissue sampling. At point C, the cutting has been completed, and the rotational speed increases again until the cutter collides with the block. After the collision, the rotational speed of the spring decreases very quickly, and then, a slight vibration is induced by the high-speed collision. However, the vibration does not influence the performance of biopsy, and the spring quickly stops rotating. Equation (5) shows that the cutting force is related to the speed before and after biopsy. After obtaining the speed at points B and C in Figure 6(c), $F_{s}$ =2.76 N is calculated according to Equation (5). It can be found that when the soft tissue is cut at high speed, the cutting force required is smaller than the static cutting force (4 N) calculated by Equation (1). Therefore, the feasibility of the high-speed soft tissue cutting scheme mentioned in this paper is further proved. In our design, the self-resetting trigger and the spiral spring is beneficial to do multiple biopsies. To test the multiple biopsies of the BCR, the high-speed camera is used to record the rotational speed of the spring. The rotational speed of the spring in biopsy is shown in Figure 6(b). The BCR can achieve continuous three times biopsies, and the maximum angular velocity of each biopsy is decreased due to the continuous release of the spiral spring energy. Moreover, the time of each biopsy is 7 ms, 7.5 ms, and 9 ms, respectively, which is related to the angular velocity. The results indicate that the BCR can achieve the multiple biopsies.

During the process of sampling, the BCR is required to reliably remove the soft tissue to avoid tearing of the soft tissue. To determine the effect of different cutting speeds on tearing, we set different cutter rotational speeds to study the tearing of soft tissue based on the research of high-speed cutting experiments. When cutting tissue, the speed of the cutter will descend due to the consumption of energy. Considering the deformation of soft tissue, the change of cutting speed is indefinite in each biopsy. So, the speed (point B) that the cutter begins to cutting the tissue is taken as the cutting speed in Figures 6(d)–6(g). Figure 6(d) shows the soft tissue sampling results at a cutting speed of 0.50 rad/ms under a 1000× electron microscope. It can be found that the sample is still attached to the colon and the tear length is about 4 mm. We tested it several times at cutting speed of 1.50 rad/ms. The soft tissue was sampled, and no tear occurred at the sampling site, as shown in Figure 6(g). Figure 6(e) shows the sampled tissue at 0.62 rad/ms, and the tear length is about 1 mm. With the change of the cutter rotational speeds, we finally found that when the cutting speed was 1.05 rad/ms, the tearing phenomenon did not occur at the sampling position of the soft tissue, as shown in Figure 6(f). Through the above analysis, it can be found that the tearing problem of soft tissue sampling can be effectively avoided by high-speed cutting of the cutter, and when the cutting speed of the cutter is 1.05 rad/ms, the soft tissue can be effectively sampled to realize biopsy.

### 4.4. In Vitro Test of the Biopsy Capsule Robot

**Figure 7:**
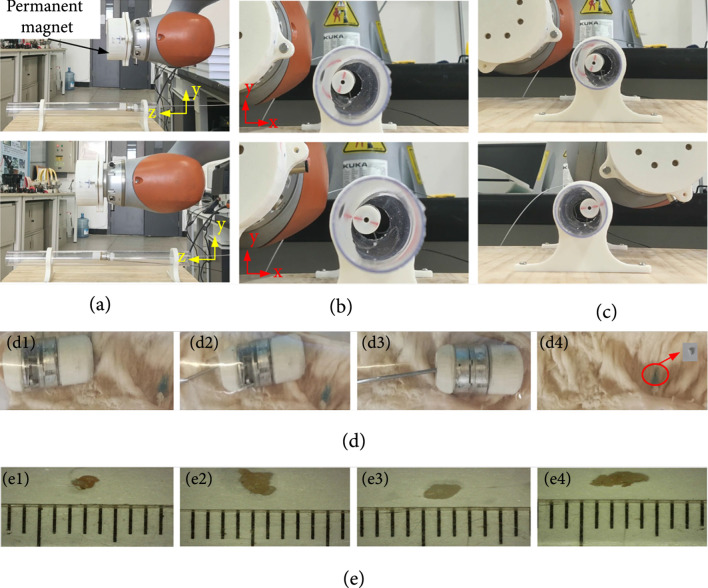
(a) The z-axis translation of BCR. (b) The rotation of BCR around self-axis. (c) The rotation of BCR around the axis of the transparent tube. (d) The locomotion of BCR in the porcine large intestine and the biopsied tissue of the porcine large intestine. (e1) Sample is approximately 2.5 ${\text{mm}}^{3}$, and the tension in the cable is 14.02 N. (e2) Sample is approximately 5 ${\text{mm}}^{3}$, and the tension in the cable is 16.32 N. (e3) Sample is approximately 4 ${\text{mm}}^{3}$, and the tension in the cable is 13.16 N. (e4) Sample is approximately 5.5 ${\text{mm}}^{3}$, and the tension in the cable is 12.67 N.

In this design, the hole of the BCR needs to be aligned with the lesion and attached to the inner wall of the intestine to complete biopsy. In order to adjust the posture of the BCR, it is necessary to test the locomotion of the BCR. We utilize KUKA robot (LBR iiwa 7 R800) with a magnet to control the posture of BCR. To verify the ability of the capsule to adjust its posture during biopsy, we placed the designed BCR in a transparent round tube, and two cameras (IMX686) were placed on the xy plane and the yz plane. A permanent magnet is installed at the end of the KUKA robot, and the BCR can move in the transparent tube driven by the permanent magnet. Figure 7(a) shows the BCR’s movement in the z-direction. The BCR rotates around its own axis above the left of the transparent tube as shown in Figure 7(b). Figure 7(c) shows that the BCR rotates around the axis of the transparent tube along the inner wall of the transparent tube. The combination of these movements allows the hole of the BCR to be aligned with the lesion location for biopsy.

A sample of suspicious tissue should be at least 1-5 ${\text{mm}}^{3}$ [22] to enable multiple diagnostic tests. In order to verify the biopsy performance of the proposed BCR, we execute an in vitro test using porcine large intestine. Figure 7(d) shows the movement of the BCR in the intestine driven by an external magnet. After reaching the target position, the operator pulls the tendon in the sheath, and the cutter in the BCR cuts the soft tissue to complete the biopsy operation. We can get the threshold of the trigger force 10.356 N calculated by the theoretical Equation (8). However, the threshold of the trigger force is ideal because of the simplified statics model. To obtain accurately the threshold, we have conducted trigger experiments ten times to obtain the threshold is 12.32 N and the typical four biopsies are shown in Figure 7(e). Figure 7(e) shows the cutting site of the tissue under a 1000× electron microscope (SHOCREX KR-001). We measure the volume of the samples under an electron microscope. Since the shape of the biopsy samples is not very regular, the estimated volumes of the samples are obtained. The smallest sample is about 2.5 ${\text{mm}}^{3}$, as shown in Figure 7(e1), and the largest sample volume is about 5.5 ${\text{mm}}^{3}$, as shown in Figure 7(e4). Hence, the cutting method can sample enough suspicious tissue to satisfy clinical biopsy requirements. The reason that the volumes of samples are different may be that the inside of the intestine is not very smooth, resulting in different contact levels between the cutter and the wall of the intestine. During four biopsies, the minimum tension in the cable is 12.67 N, and the maximum tension is 16.32 N. This discrepancy could be attributed to the friction forces of the trigger system. It can also be found that there is no relationship between the trigger force and the sampling volume, which can be also explained by the theoretical method.

### 4.5. Discussion

Although the BCR can effectively achieve biopsy, there is a challenge for biopsy visualization. The camera is located on the front of the BCR, and the rotational cutter moves in the radial direction of the BCR. When the BCR reaches the biopsy site under the control of external magnetic field, the BCR will lose the field of vision of biopsy. However, with the development of the simultaneous localization and mapping about surgical environments [23], the 3D map of colon can be constructed as the BCR moves, and the location and angle of the BCR can be recorded in the map. This map can assist the operator to align the hole of the BCR with lesion site. Furthermore, we hope to install another camera to observe the biopsy site to ensure reliable biopsy in the future work.

## 5. Conclusions

The CR is a promising alternative to the endoscopic method for gastrointestinal diagnosis because of its low discomfort for users. However, most CRs are only capable of image acquisition and lack the ability to take samples of suspicious tissues, because it is challenging to design BCRs that can acquire intestinal tissues without causing tearing or adhesion due to the flexibility of colonic tissues. In this study, we design a BCR with a novel sampling strategy in which sharp blades are rotated at high speed to scratch the intestinal wall and thereby acquire samples without tissue tearing. This BCR can achieve effective and reliable sampling because the cutting force that the force required by the cutter to cut the soft tissue is low and the tissue deformation is small. A small permanent magnet is installed in the BCR, and the locomotion of the BCR can be controlled by the external magnetic field. A spiral spring with high energy density is designed and used to power blade rotation instead of a motor or magnet. The rotational motion from the spiral spring is transmitted to the blades via a gear mechanism. To guarantee reliable sampling, a cable-sheath mechanism is used to trigger sampling performance, and the tension in the cable can be monitored in real time by a force sensor installed on the site of the manipulator. A prototype of the BCR is designed and fabricated, and its performance is verified through in vitro experiments. The results show that the proposed sampling strategy is effective and the size of acquired samples satisfies clinical requirements. We plan to integrate an image capturing function into the BCR and test locomotion of the BCR in in vivo under an external magnetic field with visual guidance.

## Data Availability

The data used to support the findings of this study are included within the article.
